# Analysis of metabolic liver function and MR-morphological cholestatic parameters after SBRT of liver metastases

**DOI:** 10.1186/s13014-025-02731-7

**Published:** 2025-10-13

**Authors:** Constantin Dreher, Paulina Wojtal, Maren Johann, Alicia S. Bicu, Lena Kaestner, Christel Weiss, Svetlana Hetjens, Anoshirwan A. Tavakoli, Dominik Nörenberg, Oliver Blanck, Hans Oppitz, Daniel Buergy, Frank A. Giordano, Judit Boda-Heggemann

**Affiliations:** 1 Department of Radiation Oncology, University Medical Center Mannheim, Medical Faculty Mannheim, University of Heidelberg, Theodor-Kutzer Ufer 1-3, 68167 Mannheim, Germany; 2 DKFZ-Hector Cancer Institute at University Medical Center Mannheim, Mannheim, Germany; 3 Mannheim Institute for Intelligent Systems in Medicine (MIiSM), University of Heidelberg, Mannheim, Germany; 4 Junior Research Group “Intelligent Imaging for Adaptive Radiotherapy”, Mannheim Institute for Intelligent Systems in Medicine (MIiSM), University of Heidelberg, Mannheim, Germany; 5 Department of Medical Statistics and Biomathematics, University Medical Center Mannheim, University of Heidelberg, Mannheim, Germany; 6 Department of Radiology and Nuclear Medicine, University Medical Center Mannheim, Medical Faculty Mannheim, University of Heidelberg, Mannheim, Germany; 7 Department of Radiation Oncology, University Medical Center Schleswig-Holstein, Kiel, Germany; 8 Saphir Radiosurgery Center Northern Germany, Kiel, Germany

**Keywords:** SBRT, Liver, Metastases, Metabolic liver function, Toxicity, MRI

## Abstract

**Background:**

This study examined the longitudinal changes in metabolic liver function and MR-morphological dilatation of intrahepatic bile ducts in patients with stereotactic body radiation therapy (SBRT) for liver metastases.

**Methods:**

This retrospective study included 64 patients with SBRT of 84 liver metastases between February 2005 and January 2019. To evaluate hepatobiliary toxicity, the laboratory parameters albumin, alanine-transaminase (ALAT), aspartate-transaminase (ASAT), bilirubin and gamma-glutamyltransferase (GGT) and the qualitative dilatation of MR-morphological peritumoural, intrahepatic bile ducts were analyzed from pre- up to 12 months post-SBRT.

**Results:**

The liver metastases were irradiated with a median D50 to the GTV of BED_α/β=10 Gy_ = 134 Gy (range 51–219 Gy) resulting in a median mean dose D_mean_-L-EQD2_α/β=3 Gy_ = 11.8 Gy (range 0.4–65.6 Gy) to the total liver. The central hepatobiliary tract (cHBT) was exposed to a median D_mean_-cHBT-BED_α/β=10 Gy_ = 8.3 Gy (range 0.1–81.6 Gy). Significant decreases in albumin and increases in GGT and bilirubin were observed up to 12 months post-SBRT. D_mean_-L-EQD2_α/β=3 Gy_, D_mean_-cHBT-BED_α/β=10 Gy_ and VBED_α/β=10Gy_66Gy-cHBT and VBED_α/β=10Gy_72Gy-cHBT were significant cofactors influencing the course of GGT, ASAT and bilirubin, but not for albumin and ALAT. MR-morphological, short- and long-term dilatation of peritumoural bile ducts were associated with significant higher VBED_α/β=10Gy_66Gy-cHBT and VBED_α/β=10Gy_72Gy-cHBT values and were significantly more frequent for SBRT of target volumes with < 3 cm distance to the cHBT.

**Conclusion:**

SBRT of liver metastases was associated with minor alterations in metabolic liver function. High dose exposure and proximity of the liver metastases to the cHBT may lead to locoregional bile duct dilatation after SBRT. Further evaluation of metabolic and MR-morphological changes in liver function is recommended in personalised oncological treatment approaches for liver-directed therapies.

## Background

The liver is one of the most frequently affected metastatic organs and liver metastases represent the most common malignant manifestation in the liver [1–4]. For a significant proportion of patients, surgical resection of liver metastases remains the standard therapeutic approach [5]. Nevertheless, due to the presence of comorbidities and the high postoperative complication rate, liver resection is not a viable option for all patients [6, 7]. Depending on individual patient factors, the location and number of metastases, surgical resection or local ablative approaches, such as stereotactic body radiotherapy (SBRT) should be considered within an interdisciplinary framework. This is particularly significant in the context of oligometastatic disease, which may allow for a curative treatment approach [8]. Consequently, comorbidities and pre-existing conditions, such as previous hepatic resection and chemotherapy, which may compromise liver function and the tolerability of liver-targeted treatment options, must be taken into account when developing personalised treatment strategies.

In the past, the use of radiation therapy for liver metastases was limited by concerns about the radiosensitivity of the liver [9–12]. However, modern, high-precision irradiation techniques such as SBRT can reduce dose exposure to radiosensitive peritumoural normal tissue [13, 14]. It is recommended that 700 cm³ of healthy liver tissue be spared from receiving >15 Gy in three fractions [11, 15], and >21.5 Gy in five fractions [16]. According to Koay et al., a mean liver dose of 15 Gy in three fractions should be achieved [17]. With regard to hepatobiliary toxicity, Osmundson et al. and Toesca et al. demonstrated the relationship between dose exposure and hepatobiliary toxicity [18, 19] - e.g. the mean dose to the cHBT (central hepatobiliary tract) as biologically effective dose with α/β = 10 Gy (D_mean_-cHBT-BED_α/β=10 Gy_) should be < 14 Gy. Considering these limitations and the technical advancements in radiation oncology, contemporary radiation techniques allow for efficacious and safe treatment options for liver cancer [9, 11, 20, 21].

The increasing use of SBRT for liver cancer, especially in combination with other treatment plans, underscores the importance of hepatic and hepatobiliary function. The effectiveness of oncologic care depends on the liver’s functional reserve, making systematic post-treatment monitoring essential.

This monitoring can be assessed from two perspectives: functional, reflecting hepatic and hepatobiliary performance through laboratory parameters and metabolic measures, and morphological, capturing structural changes on imaging. Studies have demonstrated a decline in metabolic liver function after SBRT for primary tumors, as well as morphologic MRI changes in parenchyma after SBRT for metastases [22–25]. These findings indicate the necessity of an integrated approach.

The present study therefore evaluates hepatic and hepatobilliary tolerance after SBRT for liver metastases by analyzing dose exposure, laboratory markers of toxicity, and morphological imaging changes. This combined strategy aims to define treatment-related effects in functional terms and to potentially enable individualized monitoring and follow-up.

## Methods

### Study design and patient characteristics

The data were evaluated retrospectively after approval by the local ethics committee of the Medical Faculty Mannheim, University of Heidelberg, Germany (2018-869R-MA).

A total of 64 patients (43 male, 21 female) with 84 liver metastases treated with SBRT in 69 treatment sessions at the University Medical Center Mannheim between February 2005 and January 2019 were included in this retrospective cohort-analysis. In 85.5%, 7.2%, 13.0% and 33.3% of the SBRT treatment sessions a chemotherapy, liver-directed intervention (radiofrequency ablation (RFA), microwave ablation, selective internal radiation therapy (SIRT)), radiotherapy of the liver and liver resection were performed prior to SBRT. None of the patients had biliary stents prior to SBRT.

The majority of liver metastases originated from colorectal cancer (50%), 11% from breast cancer, 10% each from pancreatic cancer or melanoma, and 19% from cancers of other origins.

The median age of the patients at the time of SBRT was 68.5 years (range 31–89 years).

### Radiotherapy

Treatment planning was performed as described previously [24]. In summary, contrast enhancement in computed tomography scan of treatment planning and deep inspiratory breath hold (Active Breathing Control (ABC), ELEKTA AB, Stockholm, Sweden) were employed [26]. Radiotherapy was delivered using a linear accelerator (Versa-HD or Synergy, ELEKTA AB). In order to enable a more accurate identification of the gross tumour volume (GTV), magnetic resonance imaging (MRI) scans were employed for treatment planning. The planning target volume (PTV) was generated from the GTV by adding a margin of 5 mm in radial direction and 10 mm in craniocaudal direction [20, 27]. Additionally, dose volume histogram (DVH) constraints for treatment planning were in accordance with the QUANTEC criteria for the surrounding organs at risk [11, 28, 29]. The radiooncological surrogate of the cHBT was contoured as described by Osmundson et al. and further assessed by Toesca et al. with 15 mm margin around the portal vein and its two main branches (for both treatment planning and retrospective analysis) [18, 19].

The proximity (< 1.5 cm vs. 1.5–3 cm vs. > 3 cm distance) of the treated target volume of SBRT to the cHBT volume was classified.

### Assessment of changes in general hepato- and hepatobiliary metabolic function and localized MR-morphology of biliary ducts

The data on the general hepato- and hepatobiliary-specific blood values (albumin, ALAT, ASAT, bilirubin and GGT) were retrospectively obtained from the clinical laboratory system and additional results were retrieved from the digital patient records. The parameters were censored after the diagnosis of hepatic progression (both in-field and out-of-field progression, which possibly influences liver function).

The blood values were collected over a period of one year following the initial fraction. The blood parameters were evaluated at approximately six weeks prior to (baseline) and six weeks following the irradiation, as well as three, six, nine and 12 months following the SBRT treatment. The blood parameters were available in 65.2 ± 7.5% at baseline, and in 48.4 ± 8.2%, 24.1 ± 5.2%, 23.5 ± 2.6%, 20.3 ± 2.3%, and 18.8 ± 3.1% approximately six weeks, three, six, nine and 12 months following the SBRT, respectively. The delta values of laboratory parameters for short-term changes (the difference between the parameters at six weeks after SBRT and six weeks prior to SBRT) and long-term changes (the difference between the parameters at one year after SBRT and six weeks prior to SBRT) were calculated. A toxicity analysis was performed using the Common Terminology Criteria for Adverse Events (CTCAE) V5.0.

The following parameters were qualitatively analyzed based on multiparametric MRI investigations conducted at different diagnostic sites with different MRI vendors at the time of treatment planning and during the follow-up period. The following parameters of localized MR-morphological biliary ducts were analyzed using T2- and T1-weighted MRI sequences and considering the radiological MRI reports: (1) pre-therapeutic local intrahepatic bile duct expansion in proximity to the liver metastases (qualitatively evaluated using a dichotomous analysis) and (2) new appearance/relative increase of expansion of local intrahepatic bile ducts during short-term follow-up (within 3 months) or (3) long-term follow-up (within 12 months). The proximity area was defined by the treated liver region. The parameters were censored after in-field hepatic progression. MRI of each metastasis was available in 96.4% at the time of treatment planning (mean − 14.8 ± 41.9 days before SBRT), 85.7% for short-term analysis (mean 68.4 ± 19.8 days after SBRT) and 52.4% for long-term analysis (mean 345.4 ± 56.7 days after SBRT).

In relation to both the general hepato- and hepatobiliary function and the localized morphological changes of the biliary ducts, the DVH parameters of treatment planning including dose prescription to the PTV PTV-BED_α/β=10Gy_ and the following hepato- and hepatobiliary DVH parameters were analyzed similarly to a former analysis regarding MR-morphological monitoring of an overlapping population [23]: 1) Liver related: The mean dose exposure to the total liver was converted to the “Equivalent Dose in 2 Gy fractions” (EQD2) with α/β = 3Gy (D_mean_-L-EQD2_α/β=3Gy_) with a cut-off point set at 18 Gy [17, 30]. Further the dose to 700ccm of the liver was converted to EQD2 with α/β = 3Gy (D700-L-EQD2_α/β=3Gy_) with a cut-off point set at 24 Gy [17]. 2) Hepatobiliary related the following parameters were assessed: The D_mean_-cHBT-BED_α/β=10Gy_, with D_mean_-cHBT-BED_α/β=10Gy_ = 14 Gy set as a cut-off point, and the volume of cHBT receiving 66 or 72 Gy as BED_α/β=10Gy_ (VBED_α/β=10Gy_66Gy-cHBT and VBED_α/β=10Gy_72Gy-cHBT) with VBED_α/β=10Gy_66Gy = 24 ccm and VBED_α/β=10Gy_72Gy = 21 ccm set as cut-off points, were investigated as described by Osmundson et al. [18].

### Statistical analysis

The longitudinal courses of metabolic blood parameters and potential cofactors were evaluated using analysis of variance (mixed model), adjusted for repeated measurements (with Scheffe adjustment for post-hoc analysis). Prior resection before SBRT, prior chemotherapy before SBRT and the DVH parameters D_mean_-L-EQD2_α/β=3 Gy_, D700-L-EQD2_α/β=3 Gy_, D_mean_-cHBT-BED_α/β=10 Gy_, and VBED_α/β=10Gy_66Gy-cHBT and VBED_α/β=10Gy_72Gy-cHBT were analyzed as potential cofactors in variance analysis.

Further, correlations of short-term and long-term changes of blood parameters with DVH-parameters were evaluated using the Pearson correlation coefficient (CC).

Significant differences of the parameters were tested using the Mann Whitney U Test. Significant differences in the distribution of ordinal/nominal parameters (relationship between target proximity to the cHBT and the MR-morphological expansion/appearance of bile ducts) were tested using the Fisher’s exact test.

The significance level was defined as a p-value below 0.05. The statistical software SAS, release 9.4 (Statistical Analysis System, SAS Institute, Cary, North Carolina, U.S.) and the R Stats Package version 4.2.1 (R Foundation for Statistical Computing) were used for analysis.

## Results

### General results

In our cohort, the median dose prescribed to the tumour was PTV-BED_α/β=10 Gy_ = 132 Gy (range 51–180 Gy). The median D98 was 115.1 Gy for the PTV-BED_α/β=10 Gy_ (range 32.8–159.2 Gy), the median D2 for the PTV-BED_α/β=10 Gy_ was 140.1 Gy (range 52.4–239.3 Gy), and the median D50 for the GTV-BED_α/β=10 Gy_ was 134.3 Gy (range 50.8–219.0 Gy). The mean dose exposure to the liver was in median D_mean_-L-EQD2_α/β=3 Gy_ = 11.8 Gy (range 0.4–65.6 Gy), the D700 of the liver was in median D700-L-EQD2_α/β=3 Gy_ = 4.7 Gy (range 0.1–57.3 Gy). In total, 22.8% and 10% of the metastases were associated with a D_mean_-L-EQD2_α/β=3 Gy_ >18 Gy and D700-L-EQD2_α/β=3 Gy_ >24 Gy. The range of D_mean_-L-EQD2_α/β=3 Gy_ and D700-L-EQD2_α/β=3 Gy_ were based on both a small liver metastasis situated in the caudal liver region and a centrally located liver metastasis. The median D_mean_-cHBT-BED_α/β=10 Gy_ of the cHBT was 8.3 Gy (range 0.1–81.6 Gy), median VBED_α/β=10Gy_66Gy-cHBT were 0.2 ccm (range 0.0–111.9 ccm) and median VBED_α/β=10Gy_72Gy-cHBT were 0.2 ccm (range 0.0–105.6 ccm). 36.5%, 14.9% and 14.9% of the metastases were associated with a D_mean_-cHBT-BED_α/β=10 Gy_ >14 Gy, VBED_α/β=10Gy_66Gy-cHBT > 24ccm, and VBED_α/β=10Gy_72Gy-cHBT > 21 ccm.

Mean liver volume at treatment planning was 1556.1 ± 390.5 cm^3^. Mean gross metastasis volume at treatment planning was 28.0 ± 47.6 cm^3^.

Taken an analogy to lung SBRT, 8% of the gross metastases were “ultracentrally” located, with direct contact or overlap with the central hepatobiliary ducts (without margin). 25% were “centrally” located within two centimeters of the central hepatobiliary ducts (without margin). 67% were more peripherally (> 2 cm) located to the central hepatobiliary ducts. Regarding the target volumes, 35%, 21%, and 44% were within 15 mm, 30 mm, or more than 30 mm of the central hepatobiliary ducts (without margin).

The 1-year local control rate and 1-year overall survival of this cohort were 83.3% and 65.4%, respectively.

### Metabolic liver function – longitudinal evaluation of laboratory values

For albumin, 6 patients developed grade I and 2 patients grade II short-term declines, while 4 patients developed grade I and 2 patients grade II long-term declines. For ASAT, 2 patients showed grade I short-term increases, with no long-term toxicity observed. For ALAT, 5 patients had grade I and 1 patient grade II short-term increases; while 2 patients developed grade I long-term increases. For bilirubin, 2 patients experienced grade I short-term increases, while 1 patient developed grade I and 1 patient grade III long-term increases. For GGT, 9 patients exhibited grade I and 1 patient grade II short-term increases, while 3 patients each developed grade I and grade II long-term increases.

**Fig. 1:**
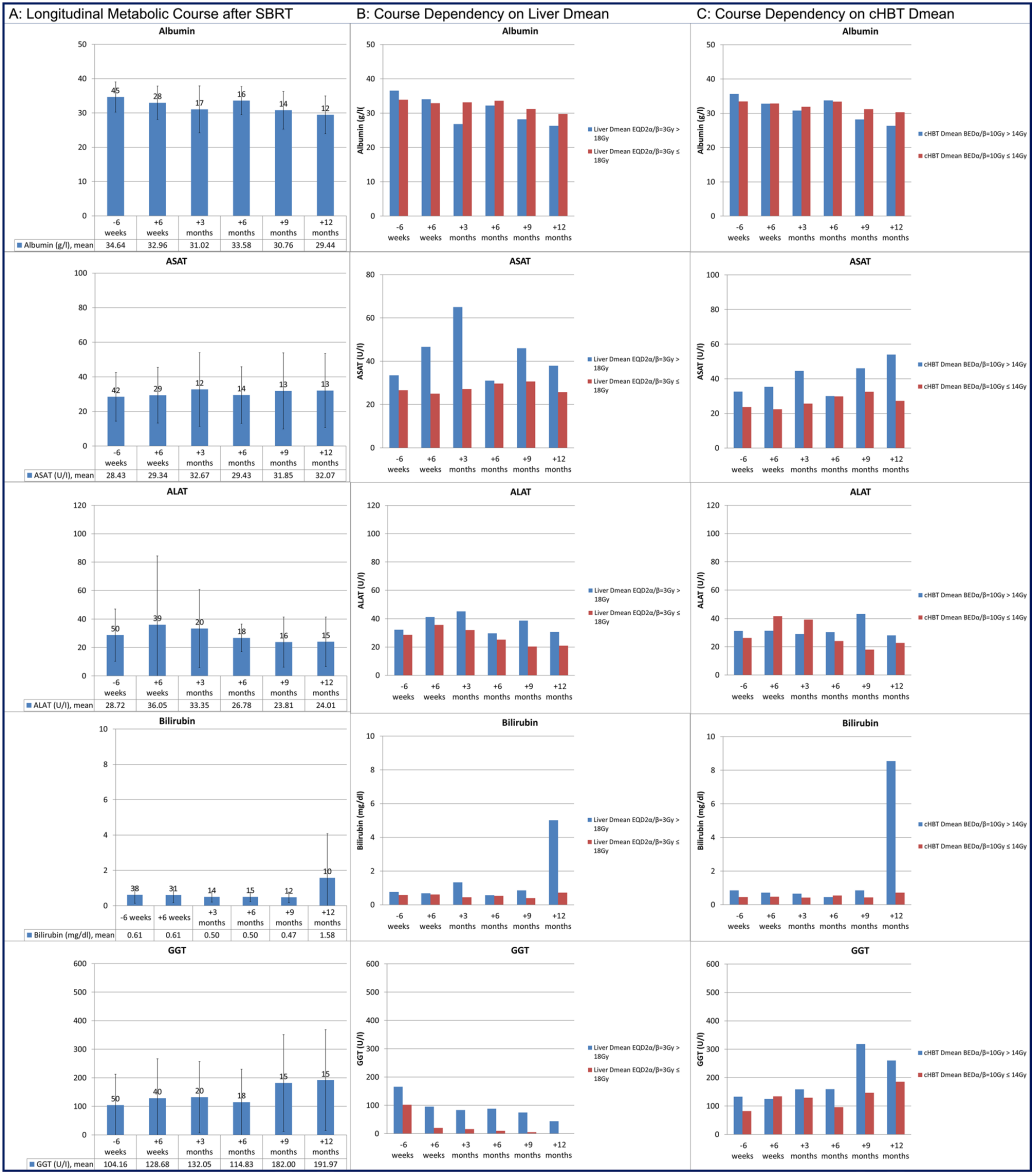
Longitudinal course of the metabolic liver function from approximately 6 weeks before SBRT of liver metastases (-6 weeks) up to 12 months after SBRT. Column A shows the mean values over time of the entire cohort, the population size is given as the numbers on top of the pillars at each time point. Dichotomized cohorts are shown in column B for liver D_mean_ EQD2 α/β = 3 Gy >/≤ 18 Gy and in column C for cHBT D_mean_ BED α/β = 10 Gy >/≤ 14 Gy

In this cohort, albumin declined (F = 4.15, *p* = 0.0023), GGT (F = 3.54, *p* = 0.0056) and bilirubin (F = 3.67, *p* = 0.0061) increased significantly up to 12 months after SBRT in analysis of variance. No significant longitudinal changes after SBRT were observed for ASAT (*p* = 0.5976) and ALAT (*p* = 0.6088). The longitudinal course of the laboratory values is demonstrated in Fig. 1.

Post-hoc analysis showed a significant difference for albumin between 6 weeks prior to and 12 months after SBRT (*p* = 0.0117), for GGT between 6 weeks prior to and 9 months after SBRT (*p* = 0.0367) and for bilirubin between 12 months after SBRT and all other time points (*p* < 0.0315). The longitudinal course is shown in Fig. 1A.

“Prior liver resection“ was only a significant cofactor for the longitudinal course of GGT (*p* = 0.023) with nominally higher GGT levels as compared to without prior liver resection. “Prior chemotherapy” was not a significant cofactor. D_mean_-L-EQD2_α/β=3Gy_ was a significant cofactor in the analysis of variance for GGT (*p* = 0.0272), ASAT (*p* = 0.003) and for bilirubin (*p* = 0.031) with nominally higher levels with D_mean_-L-EQD2_α/β=3Gy_ >18 Gy as compared to ≤ 18 Gy (Fig. 1B). D700-L-EQD2_α/β=3Gy_ was a significant cofactor in the analysis of variance for ASAT (*p* = 0.045) and bilirubin (*p* = 0.003) with higher levels with D700-L-EQD2_α/β=3Gy_ >24 Gy up to 3 months post SBRT for ASAT and up to 12 months for bilirubin (except for 6 months post SBRT).

Short-term delta-albumin correlated negatively with D_mean_-L-EQD2_α/β=3 Gy_ (CC=-0.4399, *p* = 0.0357, *n* = 23) and D700-L-EQD2_α/β=3 Gy_ (CC =-0.4237, *p* = 0.0494, *n* = 22). Long-term delta-albumin did not correlate with either D_mean_-L-EQD2_α/β=3 Gy_ or D700-L-EQD2_α/β=3 Gy_. Short-term delta-ASAT correlated positively with D_mean_-L-EQD2_α/β=3 Gy_ (CC = 0.5859, *p* = 0.0084, *n* = 19) and with D700-L-EQD2_α/β=3 Gy_ (CC = 0.5381, *p* = 0.026, *n* = 17). Long-term delta-ASAT did not correlate with D_mean_-L-EQD2_α/β=3 Gy_ or D700-L-EQD2_α/β=3 Gy_. There was no correlation of either D_mean_-L-EQD2_α/β=3 Gy_ or D700-L-EQD2_α/β=3 Gy_ with short-/long-term delta-ALAT, and –bilirubin, and short-term-delta-GGT. Long-Term-delta-GGT did correlate with D700-L-EQD2_α/β=3 Gy_ (CC = 0.6984, *p* = 0.036, *n* = 9) but not with D_mean_-L-EQD2_α/β=3 Gy_.

In the analysis of variance of longitudinal laboratory course, D_mean_-cHBT-BED_α/β=10 Gy_ was a significant cofactor for bilirubin (*p* < 0.001), GGT (*p* = 0.0060) and ASAT (*p* < 0.001), but neither for albumin nor for ALAT. The proportion of the cohort with D_mean_-cHBT-BED_α/β=10 Gy_ >14 Gy was characterized by nominally higher bilirubin, GGT and ASAT values, but not for albumin and ALAT (Fig. 1C).

Correspondingly, VBED_α/β=10Gy_66Gy-cHBT and VBED_α/β=10Gy_72Gy-cHBT were significant cofactors for bilirubin (*p* < 0.0001 and *p* = 0.0002), GGT (*p* = 0.0345 and 0.0119), and ASAT (*p* = 0.0117 and 0.0143), but neither for albumin nor for ALAT. The proportion of the cohort with VBED_α/β=10Gy_66Gy-cHBT > 24 ccm and VBED_α/β=10Gy_72Gy-cHBT > 21 ccm was characterized by nominally higher GGT values during the longitudinal course and also nominally higher ASAT and bilirubin values during short-term analysis.

D_mean_-cHBT-BED_α/β=10 Gy_, VBED_α/β=10Gy_66Gy-cHBT and VBED_α/β=10Gy_72Gy-cHBT correlated significantly with short-term delta-ASAT (CC = 0.5200, *p* = 0.0324, *n* = 17; CC = 0.4895, *p* = 0.0461, *n* = 17; and CC 0.6119, *p* = 0.009, *n* = 17) but not with long-term delta-ASAT. Additionally, VBED_α/β=10Gy_66Gy-cHBT and VBED_α/β=10Gy_72Gy-cHBT correlated significantly with short-term delta-GGT (CC = 0.5084, *p* = 0.0226, *n* = 31; and CC=-0.4044, *p* = 0.0240, *n* = 31), but not with long-term delta-GGT. There was no significant correlation with short-/long-term delta-bilirubin, -albumin, and -ALAT.

### Dependency of peritumoural MR-morphological intrahepatic bile duct expansion on metabolic liver function and dose exposure

**Fig. 2:**
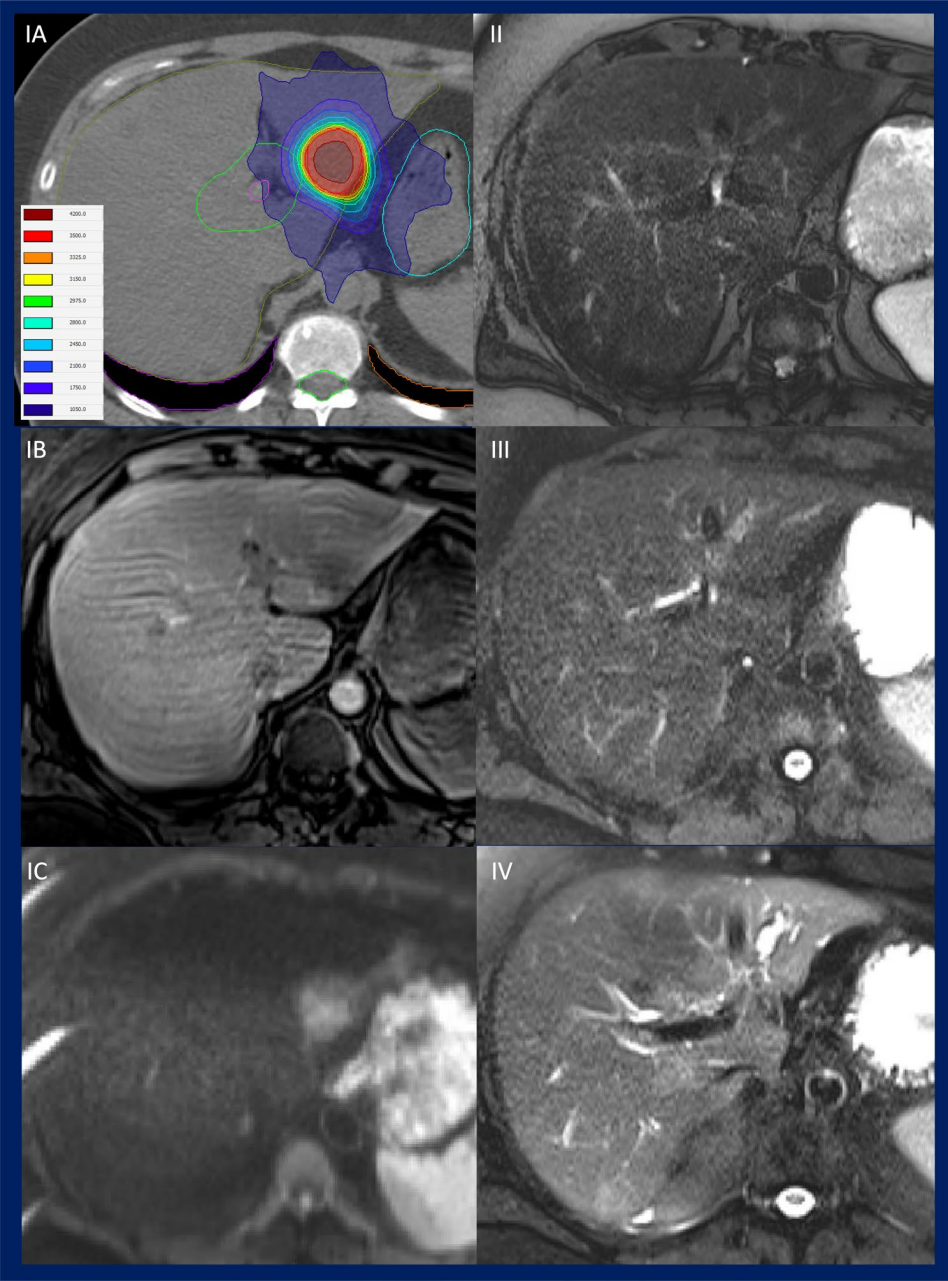
The figure depicts an exemplified longitudinal course of bile duct expansion with the area of the liver shown at approximately the same height: Left column: Treatment planning CT scan for SBRT of a liver metastasis in the left liver lobe with indication of the PTV (red) and the left part of the cHBT (green) within the liver (dark yellow) (IA). Treatment planning MRI scan with a contrast enhanced T1-weighted sequence (IB) and diffusion-weighted MRI (IC). Right column: Longitudinal course of an increase of peritumoural bile duct dilatation, as observed on T2-weighted MRI scans taken at the time of treatment planning (II), at the follow-up three months after SBRT (III) and at the 12-months follow-up after SBRT (IV)

The pre-treatment existence and short-term / long-term increase/appearance of peritumoural intrahepatic bile duct expansion have been observed in 7.4%, 8.5% and 16.3% of treated liver metastases with MRI follow-up examinations (Fig. 2).

The D_mean_ cHBT-BED_α/β=10 Gy_ was significantly elevated in instances of local cholestasis (median 5.5 Gy (range 0.1–81.6 Gy) vs. 25.8 Gy (range 15.6–72.4 Gy), *p* = 0.0097), and non-significantly in case of short-term increase/appearance of cholestasis and long-term increase/appearance of cholestasis.

VBED_α/β=10Gy_66Gy-cHBT was non-significantly elevated for local cholestasis, significantly elevated in case of short-term increase/appearance of cholestasis (*p* = 0.0290) with median 10.9 ccm (range 0.1–48.7 ccm) vs. 0.0ccm (range 0.0–111.9 ccm), and significantly elevated in case of long-term increase/appearance of cholestasis (*p* = 0.0099) with median 4.2ccm (range 0.1–111.9 ccm) vs. 0.0ccm (range 0.0–69.7 ccm).

VBED_α/β=10Gy_72Gy-cHBT was non-significantly elevated for local cholestasis, significantly elevated in case of short-term increase/appearance of cholestasis (*p* = 0.0184) with median 9.1 ccm (range 0.1–45.7 ccm) vs. 0.0 ccm (range 0.0–105.6 ccm)), and significantly elevated in case of long-term increase/appearance of cholestasis (*p* = 0.0052) with median 3.1 ccm (range 0.0–105.6ccm) vs. 0.0 ccm (range 0.0–45.7ccm).

The D_mean_-L-EQD2_α/β=3 Gy_ and D700-L-EQD2_α/β=3 Gy_ were not significantly associated with pre-existence and longitudinal increase/appearance of local intrahepatic bile duct expansion.

The distribution of pre-existing bile duct expansion was not significantly different in relation to proximity of the treated SBRT target volume to the cHBT (distances < 1.5 cm (12.0%), and 1.5–3 cm (6.7%), and > 3 cm (6.3%), *p* = 0.8571). With regard to the follow-up investigations, the appearance or relative increase of bile duct expansion was significantly more frequent in cases where the target volume was in proximity to the cHBT, both in the short-term analysis (< 1.5 cm (10.0%) vs. 1.5–3 cm (23.1%) vs. >3 cm (0.0%), *p* = 0.0152) and in the long-term analysis (< 1.5 cm (30.0%) vs. 1.5–3 cm (57.1%) vs. >3 cm (0%), *p* = 0.0004).

The pre-existence of local intrahepatic bile duct expansion was found to be independent of short- and long-term delta-bilirubin, -GGT, -ASAT, -ALAT and -albumin.

The short-term and long-term increase/appearance of local intrahepatic bile duct expansion were found to be independent of short-term delta-bilirubin (long-term delta-bilirubin was not analysed due to small cohort size) and short- and long-term delta-GGT, -ASAT, -ALAT and -albumin. However, there was a non-significant increase of long-term delta-GGT in case of long-term increase/appearance of bile duct expansion (median 4.0 U/I (range − 161.0–63.5 U/I) vs. 157.0 U/I (range 124.0–190.0 U/I), *p* = 0.09524).

## Discussion

This retrospective cohort analysis revealed minor alterations in metabolic liver function following SBRT of liver metastases. The course of GGT, ASAT and bilirubin was found to be dependent on dose exposure to the liver and the central hepatobiliary tract. Additionally, GGT was observed to be dependent on prior liver resection.

This is consistent with prior research about the dependency of RILD on significant radiation dose exposure to the liver [31] and with prior investigations in SBRT of primary liver cancer, demonstrating minor metabolic toxicity after radiotherapy [22]. However, in contrast to the investigations of Dreher et al. with SBRT of primary liver cancer and Gkika et al. with re-irradiation in primary and secondary liver cancer [22, 32], this analysis was capable of detecting multiple changes of metabolic liver function after SBRT of liver metastases. Similarly to the findings of Barry et al., the decline in albumin levels following SBRT was significantly dependent on the dose exposure to the liver [33]. Additionally, in contrast to our findings, Moon et al. reported a high frequency of elevated ASAT levels following SBRT of a mixed cohort of primary and secondary cancer [34].

Therefore, patient-specific parameters have to be taken into account, as they may potentially influence the individual course of metabolic function following locally ablative radiotherapy of secondary liver cancer. This is of particular significance, as secondary liver cancer is typically not associated with preexisting liver disease with secondary impairment of liver function. However, the primary influencing factors may be multidisciplinary oncologic treatments (e.g. prior resection) and treatment related toxicity: prior resection and high dose exposure to the liver may be influencing the course of metabolic laboratory parameters in this cohort.

Given the pronounced impact of significant alterations in laboratory findings on long-term FU after radiotherapy, it may be necessary to implement multiparametric long-term monitoring to detect long-term toxicity. In contrast to the demonstrated results of metabolic liver function, MR-morphological changes in the tumour-surrounding liver tissue mainly appeared during short-term FU and decreased in volume during long-term FU, as demonstrated by Dreher et al. in an overlapping cohort [23].

The results of this study indicate minor alterations in metabolic liver function with a significant elevation of GGT and bilirubin, suggesting that subtle cholestatic effects may have occurred following radiotherapy. These findings are supported by the detection of a corresponding MR-morphological change, as qualitative analysis of treatment planning and follow-up MRI scans revealed bile duct expansion in a small proportion of this collective. However, there was no significant dependency on metabolic liver function. The mean dose exposure to the cHBT was only significantly increased regarding pre-SBRT bile duct expansion and not significantly regarding bile duct expansion after SBRT. Therefore, dose exposure may not be the sole influencing factor for bile duct expansion. The location of the applied radiation dose to the metastases in proximity to the cHBT may be a contributing factor in the appearance or increase of bile duct expansions. However, it should be noted, that the analysis was conducted qualitatively, rather than quantitatively, which represents a significant limitation. Moreover, the analysis was primarily conducted on T2-weighted MRI scans with varying setting parameters (e.g. slice thickness, fat suppression) and from different vendors, but not by standardized, conventional ultrasound or contrast enhanced CT-scans, which are commonly employed methods for quantitative bile duct analysis in daily clinical practice. As central bile ducts are often larger in size, an increased detectability of bile duct expansions centrally, in proximity to the cHBT, may be influencing the aforementioned demonstrated connection. Furthermore, there was no significant association with metabolic liver function in our cohort, which limits the translatability of these findings to clinical cholestasis. However, in view of the observed trends, further dose assessments are recommended to facilitate the development of safe and effective cHBT dose constraints and to avoid potential toxicity [19, 35].

The study is limited by its retrospective character and the heterogeneous nature of the tumour group under analysis, which may have resulted in differing reaction to SBRT [36]. Additionally, the study is limited by the small size of the cohort, missing values due to the retrospective study design, the limited time span of long-term follow-up analysis and the fact that a proportion of the cohort has already been evaluated with regard to prognostic parameters and morphological alterations in follow-up MRI scans [24, 26]. Furthermore, some patients underwent more than one course of SBRT for liver metastases, which may introduce bias into this retrospective analysis. Moreover, the patients included in this analysis were treated in a multidisciplinary concept, which makes it challenging to discern the possible, combined effect of the various treatment regimens (including chemotherapy after radiotherapy), especially with regard to long-term follow-up, although major, metabolic and morphological changes could then occur. When evaluating DVH constraints, it is important to consider that only a very small proportion of treatment plans included in this study did not meet the constraints (especially the D700 constraint for the liver and VBED_α/β=10Gy_66Gy and 72 Gy for the cHBT). This could affect the statistical analysis of the comparison between the different population groups. In addition, the contouring of the risk structures examined (liver and especially cHBT) must be listed as a possible source of bias. In particular, the cHBT with a 15 mm margin is a surrogate structure which, although clinically investigated by Osmundson et al. and Toesca et al. [18, 19], should be further validated and possibly optimized in terms of clinical precision in a patient-specific setting. Furthermore, the censoring of systemic metabolic parameters upon systemic progression in the liver, in conjunction with the censoring of bile duct dilatation parameters upon local in-field progression in the liver, may potentially introduce a bias. Moreover, the distribution of the localization of the tumors or target volumes should be considered as a possible influencing factor. Future studies should specifically examine the subgroup with “central/ultra-central” liver metastases with regard to cHBT toxicity.

Finally, the results of the study are primarily intended to generate hypotheses. Our findings suggest several hypotheses that could be relevant for daily practice, helping radiation oncologists prevent liver function deterioration or MRI changes in their daily clinical work. For example, increases in GGT may be associated with evolving local cholestasis in proximity to the treated metastasis, linking laboratory and imaging findings. Secondly, it is important to note that bile duct dilatation occurs more frequently when lesions are located near (< 1.5 cm) the central hepatobiliary tract. This underscores the necessity to establish a definition of the location as a critical risk assessment in future analyses. This is analogous to the approach of lung stereotactic body radiation therapy (SBRT) in ultracentral/central positions. These insights support the implementation of enhanced biochemical–radiologic monitoring and more stringent central duct protection measures to avert liver function deterioration following SBRT. These possible associations require prospective validation to ensure generalizability and comparison with different liver-directed treatment approaches.

## Conclusion

Clinical practice could benefit from more individualized and closer monitoring, potentially paving the way for the development of truly personalized treatment plans. Our analysis revealed only minor alterations in metabolic liver function and provided valuable insights into the effects of SBRT with high dose of irradiation to small areas within the liver on MR-morphological bile duct expansion. Importantly, this study is the first to identify a connection between hepatic SBRT in proximity to the central hepatobiliary tract and the appearance of MR-morphological cholestatic changes.

As the question of which patients derive the greatest benefit from SBRT as a local ablative treatment approach in liver cancer remains unanswered, further investigations into individual tolerability are essential. The identified influencing factors should be further evaluated to ascertain their predictive value for toxicity. This may enable the optimisation of radiotherapy within multidisciplinary treatment concepts for both primary and secondary liver cancer.

## Data Availability

The data used and generated in this study may be made available, subject to ethical and data protection considerations, upon reasonable request on an individual basis.

## Abbreviations

ABC: Active Breathing Control
ALAT: Alanine-transaminase
ASAT: Aspartate-transaminase
BED_α/β=10 Gy_: Biologically effective dose with α/β = 10 Gy
cHBT: Central hepatobiliary tract
CTCAE: Common Terminology Criteria for Adverse Events
CC: Correlation coefficient
DVH: Dose volume histogram
EQD2: Equivalent Dose in 2 Gy fractions
D_mean_: Mean dose
GGT: Gamma-glutamyltransferase
GTV: Gross tumour volume
MRI: Magnetic resonance imaging
PTV: Planning target volume
RFA: Radiofrequency ablation
SIRT: Selective internal radiation therapy
SBRT: Stereotactic body radiation therapy
